# Highly polymorphic mitochondrial DNA and deceiving haplotypic differentiation: implications for assessing population genetic differentiation and connectivity

**DOI:** 10.1186/s12862-019-1414-3

**Published:** 2019-04-18

**Authors:** S. Fourdrilis, T. Backeljau

**Affiliations:** 1 Royal Belgian Institute of Natural Sciences, Rue Vautier 29, B-1000 Brussels, Belgium; 2 Evolutionary Ecology Group, University of Antwerp, Universiteitplein 1, B-2610 Antwerp, Belgium

**Keywords:** Differentiation statistics, Gene flow, *Melarhaphe neritoides*, Migrate-n, mtDNA hyperdiversity, Mutation rate

## Abstract

**Background:**

Hyperdiverse mtDNA with more than 5% of variable synonymous nucleotide sites can lead to erroneous interpretations of population genetic differentiation patterns and parameters (φ_ST_, D_EST_). We illustrate this by using hyperdiverse mtDNA markers to infer population genetic differentiation and connectivity in *Melarhaphe neritoides*, a NE Atlantic (NEA) gastropod with a high dispersal potential. We also provide a recent literature example of how mtDNA hyperdiversity may have misguided the interpretation of genetic connectivity in the crab *Opecarcinus hypostegus*.

**Results:**

mtDNA variation surveyed throughout the NEA showed that nearly all *M. neritoides* specimens had haplotypes private to populations, suggesting at first glance a lack of gene flow and thus a strong population genetic differentiation. Yet, the bush-like haplotype network, though visually misleading, showed no signs of phylogeographic or other haplotype structuring. Coalescent-based gene flow estimates were high throughout the NEA, irrespective of whether or not mtDNA hyperdiversity was reduced by removing hypervariable sites.

**Conclusions:**

*Melarhaphe neritoides* seems to be panmictic over the entire NEA, which is consistent with its long-lived pelagic larval stage. With hyperdiverse mtDNA, the apparent lack of shared haplotypes among populations does not necessarily reflect a lack of gene flow and/or population genetic differentiation by fixation of alternative haplotypes (D_EST_ ≈ 1 does not a fortiori imply φ_ST_ ≈ 1), but may be due to (1) a too low sampling effort to detect shared haplotypes and/or (2) a very high mutation rate that may conceal the signal of gene flow. Hyperdiverse mtDNA can be used to assess connectivity by coalescent-based methods. Yet, the combined use of φ_ST_ and D_EST_ can provide a reasonable inference of connectivity patterns from hyperdiverse mtDNA, too.

## Background

Assessing the amounts and patterning of spatio-temporal genetic diversity within and among populations provides essential information on the evolutionary dynamics of population structuring and speciation. Yet, very large amounts of genetic variation, i.e. genetic hyperdiversity where variable synonymous nucleotide sites can exceed 5%, may bias population genetic interpretations, due to the complex relationship between the statistics used to estimate genetic differentiation and processes that produce genetic differentiation [1, 2]. Genetic hyperdiversity is more common than currently appreciated and occurs in at least 43% of animal species [3]. Genetic markers differ in their levels of variability, for example nuclear DNA microsatellites are often genetically very variable [4, 5], while mitochondrial DNA (mtDNA) usually reveals more moderate amounts of genetic variation. Nevertheless, the marine, rock-dwelling, planktonic-dispersing gastropod *Melarhaphe neritoides* (Linnaeus, 1758) (Gastropoda: Littorinidae) shows hyperdiverse mtDNA [3].

Planktonic dispersers spread as planktonic larvae during early life stages and subsequently become sedentary after settlement. Planktonic dispersers with a long pelagic larval duration (PLD), such as *M. neritoides* (PLD = 4 to 8 weeks) [6], are expected to display long-distance dispersal and high rates of gene flow, and to show little, if any, population genetic differentiation even over thousands of kilometres [3, 6–8]. However, at least three methodological issues may blur the exploration of these paradigmatic expectations in species with hyperdiverse mtDNA: (1) Highly variable genetic markers whose mutation rate (*μ*) is similar or higher than the levels of gene flow, i.e. *N*_*e*_*μ* ≥ *N*_*e*_*m*, violate the assumption of a negligible *μ* [9–12]. This results in a low F_ST_ (and relatives) values that reflect the influence of mutation [F_ST_ = 1/(1 + 4*N*_e_*μ)] instead of the influence of migration [F_ST_ = 1/(1 + 4*N*_e_*m)]. Indeed, although mutations lead to genetic differentiation among populations and hence increase F_ST_, high *μ* generates numerous private alleles with low frequency as well as high within-population genetic diversity, which are two quantities that may bias genetic differentiation estimates in terms of F_ST_. On the one hand, F_ST_ is restricted to values much less than 1 (mean maximum F_ST_ ≈ 0.3585) if the frequency of the most frequent allele is low (near zero) or high (near 1) [13–16]. On the other hand, F_ST_ drops to zero when within-population diversity is high [1, 2, 17–33]. (2) Conversely, highly variable genetic markers may deceivingly suggest population genetic differentiation by concealing gene flow, because high *μ* may provoke a shortfall of shared haplotypes among populations and/or require unrealistic sample sizes to detect shared haplotypes [3]. (3) F_ST_-based methods may produce biased estimates of population genetic connectivity with highly variable markers [11, 18, 21, 26, 32–34]. In contrast, gene genealogy-based methods, such as implemented in the Migrate-n software, are suited to accommodate highly polymorphic data and produce reliable population genetic connectivity estimates over the whole spectrum of mutation rates, because the coalescent process is not dependent on mutation rates [34–37]. Gene genealogy-based methods use raw sequence data, to estimate genealogies and convert coalescent times between pairs of alleles into amounts of gene flow that would result in a similar distribution of alleles in gene genealogies. Therefore, gene genealogy-based methods can be used to assess the influence of high *μ* on estimates of population genetic differentiation inferred from frequency-based parameters.

*Melarhaphe neritoides* has hyperdiverse mtDNA with an extremely high haplotype diversity (*Hd* = 0.999 ± 0.001) and a high neutral nucleotide diversity (*π*_*syn*_ = 6.8%) for 16S, COI and Cyt*b* in the Azores [3], and for COI (*Hd* = 0.998; *π*_*syn*_ = 7.6%) at the Galician coast [38]. This is mainly explained by an extremely high mtDNA mutation rate (*μ* = 1.99 × 10^− 4^ mutations per nucleotide site per generation or 5*.*82 × 10^− 5^ mutations per nucleotide site per year, at the COI locus) [3]. The species is distributed throughout the North East Atlantic (NEA), from Southern Norway to the Canary Islands i.e. over approx. 4000 km, and even up to 5500 km if the Cape Verde Islands are included [39–41], and over a West-East beeline distance of 6000 km from the Azores in the Atlantic to Lebanon in the eastern Mediterranean and into the Black Sea [42–44]. Within the Azores, *M. neritoides* revealed no shared mtDNA haplotypes among populations [3], thus deceivingly suggesting a lack of gene flow. Yet, in view of the confounding issues listed above, it is necessary to assess the influence of mtDNA hyperdiversity in *M. neritoides* on this observation and to check how patterns of population genetic structuring in *M. neritoides* are manifested over larger geographic scales. Hence, the present study aims at exploring to what extent the hyperdiverse mtDNA of *M. neritoides* influences the assessment of population genetic differentiation and connectivity in this species. It does so by: (1) assessing mtDNA differentiation among populations at several spatial scales within the range 1–6000 km to test for panmixis throughout the NEA, (2) comparing scenarios of gene flow among three oceanographic areas in the distribution range of *M. neritoides*, viz. the Azores, the NEA coast and the Mediterranean Sea, and quantifying coalescent-based gene flow among these oceanographic areas, and (3) illustrating the influence of mtDNA hyperdiversity in the estimation of population genetic differentiation and connectivity, by comparing estimates of population genetic differentiation and gene flow using mtDNA data with different amounts of polymorphism.

## Results

### mtDNA diversity

With 30% polymorphic sites in the total population (original hyperdiverse dataset A), the mtDNA in *M. neritoides* is highly polymorphic (Table 1). Haplotype and nucleotide diversities are very high when the 11 sampling sites are pooled (*Hd* = 0.999 ± 0.001; *π* = 0.013 ± 0.001), but also at each sampling site (*Hd* = 0.993 ± 0.021 to 1.000 ± 0.005–0.008; *π* = 0.012 to 0.014 ± 0.001). The 399 individuals sequenced involved 390 different haplotypes (*H* = 390), 386 of which were private (99%). Hyperdiversity, i.e. nucleotide diversity at synonymous sites, which reflects neutral polymorphism shaped by the balance between mutation pressure and genetic drift, is observed in COI (*π*_*syn*_ = 0.0725 = 7.25%), Cyt*b* (*π*_*syn*_ = 0.0657 = 6.57%), and the concatenated dataset A when the 11 sampling sites are pooled (*π*_*syn*_ = 0.0686 = 6.86%) or at each sampling site (*π*_*syn*_ = 0.0616 to 0.0749 = 6.16 to 7.49%). For 16S, *π*_*syn*_ is not applicable because this gene fragment is not protein-coding and thus has no synonymous and non-synonymous sites. In contrast, non-neutral polymorphism is low (*π*_*nonsyn*_ = 0.0005 = 0.05% maximum). COI, Cyt*b* and 16S all show high levels of haplotype diversity (*Hd*_COI_ = 0.995 ± 0.001; *Hd*_Cyt*b*_ = 0.998 ± 0.001; *Hd*_16S_ = 0.848 ± 0.001), proportion of polymorphic sites (*S*_COI_ = 33%; *S*_Cyt*b*_ = 34%; *S*_16S_ = 22%) and of private haplotypes (89.6% in COI; 89.3% in Cyt*b*; 77.2% in 16S), although the 16S haplotypes differ from each other only by single nucleotides (*π* = 0.004 ± 0.001).

**Table 1:** Genetic diversity in *Melarhaphe neritoides* in the North East Atlantic

	*N*	*H*	*H*_*p*_	*H*_*s*_	*H*_*w*_	*L*	*S*	*Hd* ± SD	*π* ± SD	*π*_*syn*_	*π*_*nonsyn*_
16S	399	145	112	33	3	486	106 (22%)	0.848 ± 0.001	0.004 ± 0.001	n/a	n/a
COI	399	309	277	32	3	614	200 (33%)	0.995 ± 0.001	0.018 ± 0.001	0.0725	0.0001
Cyt*b*	399	328	293	35	6	675	230 (34%)	0.998 ± 0.001	0.016 ± 0.001	0.0657	0.0005
16S-COI-Cyt*b* (dataset A)											
Total population	399	390	386	4	1	1775	536 (30%)	0.999 ± 0.001	0.013 ± 0.001	0.0686	0.0003
FAI	42	42	42	0	0	1775	205 (12%)	1.000 ± 0.005	0.013 ± 0.001	0.0749	0.0003
FLO	39	39	37	2	0	1775	183 (10%)	1.000 ± 0.006	0.012 ± 0.001	0.0620	0.0005
PIC	37	36	34	3	1	1775	185 (10%)	0.998 ± 0.007	0.012 ± 0.001	0.0616	0.0003
POR	38	38	37	2	0	1775	173 (10%)	1.000 ± 0.006	0.012 ± 0.001	0.0636	0.0003
RHO	39	39	38	1	0	1775	187 (11%)	1.000 ± 0.006	0.013 ± 0.001	0.0661	0.0002
SCO	18	17	15	4	0	1775	120 (7%)	0.993 ± 0.021	0.013 ± 0.001	0.0706	0.0000
SM1	35	35	35	0	0	1775	210 (12%)	1.000 ± 0.007	0.013 ± 0.001	0.0673	0.0004
SM2	37	37	36	1	0	1775	195 (11%)	1.000 ± 0.006	0.013 ± 0.001	0.0692	0.0001
SM3	43	43	43	0	0	1775	239 (14%)	1.000 ± 0.005	0.014 ± 0.001	0.0728	0.0003
SMA	32	32	32	0	0	1775	217 (12%)	1.000 ± 0.008	0.014 ± 0.001	0.0708	0.0005
SPA	39	38	36	4	0	1775	183 (10%)	0.999 ± 0.006	0.014 ± 0.001	0.0729	0.0002
16S-COI-Cyt*b* (dataset B)											
Total population	399	161	134	27	3	1429	191 (13%)	0.824 ± 0.001	0.001 ± 0.001	n/a	n/a

Dataset A is the original hyperdiverse mtDNA dataset. Dataset B is the same, but with the most variable nucleotide sites removed (see text). *N*, number of individuals; *H*, number of haplotypes; *H*_*p*_, number of private haplotypes; *H*_*s*_, number of haplotypes shared among sampling sites; *H*_*w*_, number of haplotypes shared within sampling site; *L*, DNA fragment length in base pairs; *S*, number of segregating sites (and in % of the fragment length); *Hd*, haplotype diversity ± standard deviation; *π*, Jukes-Cantor corrected nucleotide diversity ± standard deviation; *π*_*syn*_, Jukes-Cantor corrected nucleotide diversity at synonymous sites; *π*_*nonsyn*_, Jukes-Cantor corrected nucleotide diversity at non-synonymous sites; n/a, not applicable. For the abbreviation of sampling site names, see Fig. 3

### mtDNA population differentiation

In the total population (original hyperdiverse dataset A), G_ST_ and φ_ST_ reveal very low, but significant differentiation (G_ST_ = 0.001, *p* = 0.02; φ_ST_ = 0.005, *p* = 0.04), whereas N_ST_ suggests no significant differentiation (N_ST_ = 0.004, *p* = 1.00). So, haplotype frequencies are usually similar among sampling sites (Table 2).

**Table 2:** Population genetic differentiation in *Melarhaphe neritoides* in the NEA based on two DNA polymorphism levels

	G_ST_	*p*	φ_ST_	*p*	N_ST_	*p*	D_EST_	*CI*
16S-COI-Cyt*b*								
dataset A	**0.001**	*0.02*	**0.005**	*0.04*	0.004	*1.00*	**0.679**	*0.664–0.688*
dataset B	0.006	*0.20*	**0.005**	*0.03*	0.004	*0.43*	0.026	*0.000–0.100*

(dataset A) original highly polymorphic 16S-COI-Cyt*b* dataset, (dataset B) modified 16S-COI-Cyt*b* dataset with reduced polymorphism. Values significantly different from zero are in bold

In contrast, Morisita’s unbiased dissimilarity index is significant (D_EST_ = 0.679, CI = 0.664–0.688) and shows strong haplotypic differentiation in the total population. This means that haplotypes are usually distinct among the 11 sampling sites, with complete haplotypic differentiation (D_EST_ = 1) in 47 of the 55 pairs of sampling sites, including the two geographically closest sites SM2 and SM3 that are 1.2 km apart. Five out of 390 haplotypes occur in more than one individual (*H*_*s*_ = 4 and *H*_*w*_ = 1) (Table 1). Since one of these haplotypes is shared by two individuals of the same sampling site (*H*_*w*_ = 1), only four haplotypes are shared among sampling sites (*H*_*s*_ = 4), viz. within AZOR (between FLO and SM2), within ATCO (among the three sampling sites), between AZOR and ATCO (among PIC, POR, SCO, SPA), and between AZOR and MEDI (between FLO and RHO). The most common haplotype (hap 108) is shared between AZOR and ATCO, with a low frequency of 0.0125 (Table 3). No haplotypes are shared between ATCO and MEDI. Therefore, of the 390 haplotypes, the vast majority is private to sampling sites (*H*_*p*_ = 386 out of 390 haplotypes) and involves 96.7% of the 399 individuals sequenced (Table 1). Four of the 11 sampling sites (FAI, SM1, SM3 and SMA), located in the Azores, share no haplotypes with other sampling sites (*H*_*s*_ = 0).

**Table 3:** Pairwise mtDNA differentiation in *Melarhaphe neritoides* among 11 sampling sites in the NEA

	FAI	FLO	PIC	POR	RHO	SCO	SM1	SM2	SM3	SMA	SPA
FAI	0										
FLO	0.006 (0.179)	0			hap 73			hap 49			
PIC	0.012 (0.070)	0.008 (0.145)	0	hap 108		hap 108					hap 108
POR	0.008 (0.122)	0.007 (0.156)	−0.005 (0.666)	0		hap 108					hap 108
RHO	0.009 (0.122)	−0.007 (0.842)	0.010 (0.120)	0.005 (0.207)	0						
SCO	**0.029 (0.006)**	0.006 (0.191)	0.017 (0.061)	**0.025 (0.018)**	0.015 (0.054)	0					hap 108 hap 201
SM1	0.009 (0.123)	−0.005 (0.739)	0.003 (0.300)	0.009 (0.119)	0.003 (0.288)	−0.002 (0.532)	0				
SM2	0.003 (0.325)	−0.011 (0.870)	−0.011 (0.781)	0.000 (0.415)	−0.001 (0.447)	0.009 (0.212)	−0.008 (0.715)	0			
SM3	0.002 (0.321)	−0.011 (0.992)	0.014 (0.063)	0.007 (0.145)	−0.006 (0.839)	0.011 (0.084)	0.001 (0.340)	−0.002 (0.519)	0		
SMA	0.003 (0.287)	0.006 (0.182)	0.010 (0.114)	0.003 (0.269)	0.008 (0.138)	**0.037 (0.003)**	**0.016 (0.042)**	0.001 (0.386)	0.002 (0.329)	0	
SPA	−0.001 (0.513)	−0.009 (0.959)	0.008 (0.134)	0.006 (0.169)	−0.005 (0.762)	0.007 (0.151)	−0.004 (0.697)	−0.011 (0.891)	− 0.009 (0.963)	0.002 (0.296)	0

Above diagonal: haplotypes shared between pairs of sampling sites. Below diagonal: φ_ST_ values (with p-values). Significant φ_ST_ values (α = 0.050) are in bold. No values remained significant after sequential Bonferroni correction for multiple test biases (α = 0.001). For the abbreviation of sampling sites names, see Fig. 3

The non-hyperdiverse mtDNA dataset B provides the same picture of low but significant (only φ_ST_) differentiation among sampling sites in the NEA (Table 2). However, haplotypic differentiation in the total population disappears (D_EST_ = 0.026, CI = 0.000–0.100, i.e. not significantly different from zero), due to the reduced mtDNA variability in dataset B and the larger proportion of shared haplotypes (17% against 1% in dataset A).

We assessed population genetic differentiation at several spatial scales within the range 1–6000 km over the NEA basin among the three oceanographic areas ATCO, AZOR and MEDI (Table 4). At large scale, over the entire NEA, the AMOVA (dataset A) shows no significant differentiation among sampling sites (φ_SC_ = 0.003, *p* > 0.05) or among the three areas AZOR, ATCO and MEDI (φ_CT_ = 0.004, *p* > 0.05) (Table 4).

**Table 4:**
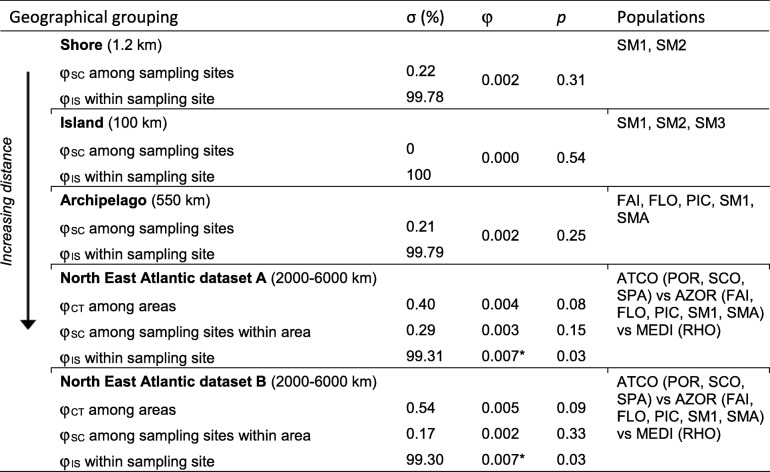
φ-based hierarchical AMOVA showing mtDNA differentiation among and within sampling sites of *Melarhaphe neritoides* in the NEA

For each AMOVA are given the spatial scale (in parenthesis), the percentage of among-group variance or within-group variance (σ), the φ-statistic (φ, significant values marked with * for *p* < 0.05) and the associated probability of significance (*p*). For the abbreviation of geographical groupings and sampling sites names, see Fig. 3.

Very low but significant differentiation is detected at the within-sampling site level using the hyperdiverse dataset A (φ_IS_ = 0.007, *p* = 0.03), which is not an artefact of mtDNA hyperdiversity since identical results are obtained using the non-hyperdiverse dataset B (φ_IS_ = 0.007, *p* = 0.03). The φ_IS_ index reflects high variation among individuals of the same sampling site (σ = 99.31%) and not differentiation among sampling sites of the three areas or among areas. Indeed, at all spatial scales, the AMOVAs show that > 99% of the variation is due to within-sampling site variation and not to among-sampling site differentiation (< 1%). Moreover, none of the pairwise large-scale area comparisons of genetic differentiation (φ_ST_) show significant values (Table 3). At smaller spatial scales, no population genetic structure is detected, neither among Azorean islands (100–550 km), nor among sampling sites on the same shore (1.2 km). Hence, these data suggest that there is no genetic differentiation among sampling sites at any scale over the entire species’ distribution range.

### Population genetic connectivity

**Fig. 1:**
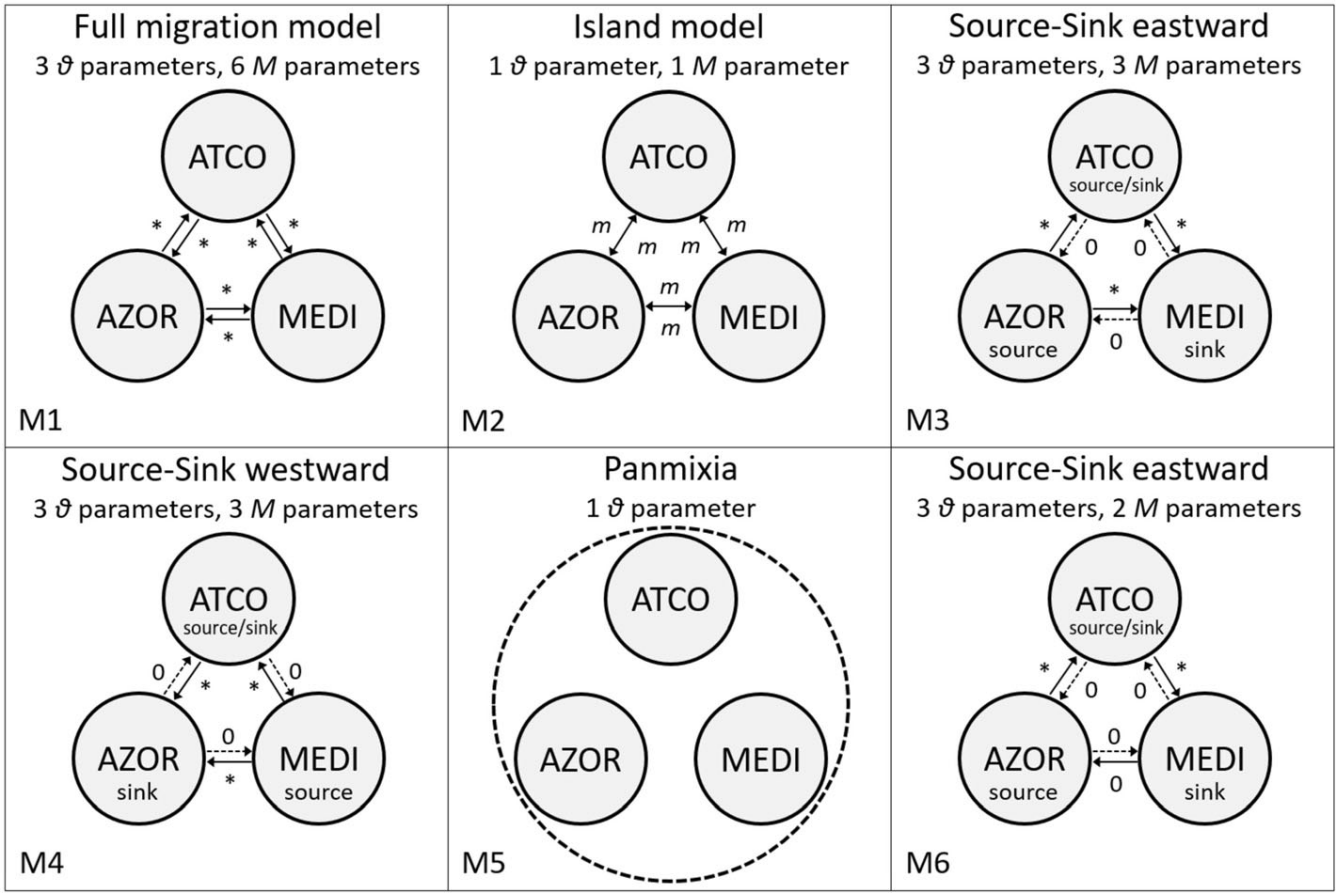
Connectivity pattern in *Melarhaphe neritoides* inferred from model M6 providing directions of gene flow. Gene flow values (*N*_*e*_*m*) are based on the hyperdiverse dataset A, and on the non-hyperdiverse dataset B (italic), with corresponding confidence interval in parenthesis. The arrows represent directions of migration among the three oceanographic areas AZOR (Azores archipelago) in yellow, ATCO (North East Atlantic coast) in pink and MEDI (Mediterranean) in blue. The thickness of arrows is proportional to the inferred rates of gene flow, and dashed line represents the absence of gene flow

Gene flow estimates with Migrate-n applied to datasets A and B suggest that *M. neritoides* complies with a panmictic model, and hence that the species behaves as a single panmictic population over its entire distribution range. Indeed, M5 has the lowest log marginal likelihood of the six gene flow models tested, and the highest probability (*p* = 0.755) (Table 5), thus explaining 75.5% of how gene flow is patterned. Based on M5, the effective population size of *M. neritoides* in the NEA is comparatively small (*N*_*e*_ = 2587, CI = 2225–2941; using *θ* = 0.51476) relative to the effective population size in the Azores (*N*_*e*_ = 5256, CI = 1312–37,495) [Cf. 3]. The panmictic model M5 does not allow to quantify separately the immigration rates among the three areas, since all sampling sites are pooled into one single population. The second model that has a non-zero probability (*p* = 0.245) is the Source-Sink eastward model (M6), which contributes to describing for 24.5% how gene flow is patterned in *M. neritoides*. Rates of gene flow among the three areas are higher eastward than westward (Fig. 1). The Mediterranean (MEDI) receives large numbers of immigrants per generation from ATCO (*N*_*e*_*m* = 4419; CI = 1499–8634; using *M*_*ATCO➔MEDI*_ = 4051.6) but not from AZOR. The ATCO area also receives large numbers of immigrants per generation from AZOR (*N*_*e*_*m* = 558; CI = 288–922; using *M*_*AZOR➔ATCO*_ = 1326.3). The other models have a near-zero (M1, M3, M4) or zero (M2) probability and hence these models are not further considered.

**Table 5:** Ranking of the gene flow models in *Melarhaphe neritoides* tested in Migrate-n

Rank	Model	log marginal likelihood	LBF	probability
mtDNA dataset A (hyperdiverse)				
1	M5 Panmixia	−20,624.08498	0.0	0.755
2	M6 Source-Sink eastward	−20,625.20809	−1.1	0.245
3	M3 Source-Sink eastward	−20,649.53419	−25.5	6.69 × 10^−12^
4	M1 Full migration model	−21,035.49377	−411.0	1.60 × 10^−179^
5	M4 Source-Sink westward	−21,051.61336	− 427.5	1.60 × 10^− 186^
6	M2 Island model	−21,655.46617	− 1031.4	0.000
mtDNA dataset B (low polymorphism)				
1	M5 Panmixia	− 2692.03118	0.0	1.000
2	M6 Source-Sink eastward	− 2799.74770	− 107.7	1.66 × 10^−47^
3	M3 Source-Sink eastward	− 2802.66687	−110.6	8.94 × 10^−49^
4	M1 Full migration model	− 2812.21261	− 120.2	6.40 × 10^−53^
5	M4 Source-Sink westward	− 2815.14498	− 123.1	3.41 × 10^−54^
6	M2 Island model	− 2887.11249	−195.1	1.89 × 10^−85^

Models are ranked using log Bayes factors (LBF) and probabilities that are based on the comparison of the log marginal likelihood of each model

Assessing genetic connectivity using the Migrate-n analysis of the non-hyperdiverse dataset B yields the same ranking of gene flow models (Table 5), in less computing time (5 to 24 days) than dataset A (18 to 34 days), but increases the model probability of the first-ranked model M5 to the maximal value (*p* = 1) and drops that of the second-ranked model M6 to near-zero (*p* = 1.66 × 10^− 47^).

Fay & Wu’s H shows significant signal of selection in 16S-COI-Cyt*b* (*Hn* = − 10.4116, CI = − 2.4382–0.9862).

**Fig. 2:**
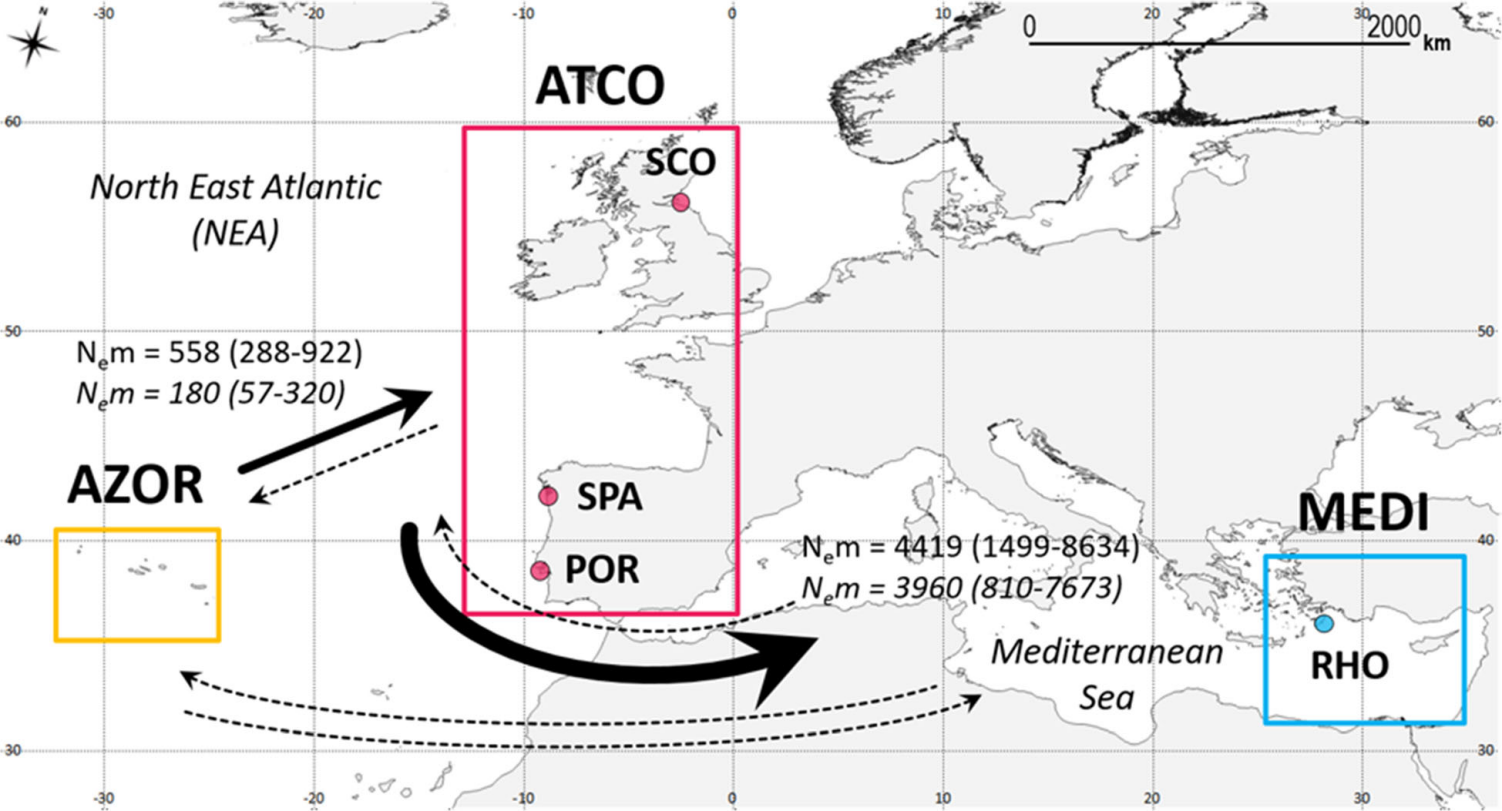
Median-joining networks of mtDNA in *Melarhaphe neritoides*. (**a**) concatenated 16S-COI-Cyt*b* (dataset A), (**b**) COI, (**c**) Cyt*b*, (**d**) 16S and (**e**) concatenated 16S-COI-Cyt*b* (dataset B). The size of circles is proportional to the number of individuals per haplotype. Haplotype origins: AZOR, Azores archipelago – yellow; ATCO, North East Atlantic coast – pink; MEDI, Mediterranean – blue

The hyperdiverse mtDNA data, i.e. the combined 16S-COI-Cyt*b* dataset A and the single COI and Cyt*b* genes, all show bush-like haplotype networks (Fig. 2 a, b, c) of private haplotypes represented by single individuals (i.e. singletons) and very few shared haplotypes among sampling sites (sectored circles), a pattern characteristic of DNA hyperdiversity. Intuitively, such pattern would not be associated with a strong signal of gene flow and population connectivity. Yet, it is exactly a pattern one would expect for high gene flow and strong connectivity [45]. Moreover, the lack of an association between haplotype relationship and geography in the networks suggests the absence of phylogeographic structure in *M. neritoides* in the NEA, which is also supported by the non-significant difference between N_ST_ and G_ST_ (N_ST_ – G_ST_ = 0.003), indicative of no phylogeographic signal [46]. The impact of mtDNA hyperdiversity becomes clear in the haplotype networks of the non-hyperdiverse combined 16S-COI-Cyt*b* dataset B and the single 16S data, showing a classic star-like pattern typical of population expansion and high gene flow [47], where most new haplotypes arise by recent mutation events from a central widespread haplotype (Fig. 2 d, e).

## Discussion

### Genetic hyperdiversity and assessing population genetic differentiation

The present study confirms that mtDNA in *M. neritoides* is hyperdiverse (*π*_*syn*_ ≥ 5%), not only in the Azores and Galicia [3], but all over the NEA. This mtDNA hyperdiversity results in an overwhelming number of private haplotypes and a paucity or lack of shared haplotypes among sampling sites as close as 1.2 km. Despite this nearly complete haplotypic differentiation (D_EST_) among sampling sites, there is no significant pairwise population genetic differentiation (φ_ST_). Yet, in the absence of hyperdiversity (dataset B), the haplotypic differentiation drops to zero, and thus showing the effect of mtDNA hyperdiversity on D_EST_. Therefore, when using hyperdiverse mtDNA markers, population genetic differentiation in terms of lack of haplotype sharing may be substantial, but is not indicative of population genetic differentiation in terms of fixation of alternative haplotypes, i.e. D_EST_ ≈ 1 does not a fortiori imply φ_ST_ ≈ 1. From a practical point of view, hyperdiverse mtDNA may require unrealistically high sampling efforts to detect haplotypes more than once and to reliably assess haplotype sharing among populations [3]. This phenomenon is best explained by the high mutation rates in hyperdiverse mtDNA, which generate numerous private haplotypes with low frequency that provoke a high within-population genetic diversity [3] influencing D_EST_, but not φ_ST_ [48].

mtDNA hyperdiversity represents the upper boundary of intra-specific genetic variation, and allowed us to use φ_ST_ and D_EST_ at a limit of their applicability, i.e. for extreme intra-population variation. mtDNA hyperdiversity reveals that φ_ST_ (and related indices such as F_ST_ and G_ST_) reliably measures population genetic differentiation in terms of dissimilarities in the frequencies of shared haplotypes and degrees of fixation of alternative haplotypes among populations, whereas D_EST_ reliably measures differentiation in terms of lack of haplotype sharing among populations. This is in accordance with the use of F_ST_ recommended by Wright [18, page 82], and the use of D_EST_ intended by Jost [26]. The two indices thus measure two different, but complementary characteristics of population genetic differentiation.

### Is Melarhaphe neritoides panmictic?

Our assessment of population genetic differentiation in *M. neritoides* in the NEA, based on mtDNA markers that are far more variable than Johannesson’s [6] allozyme data, confirms that the pattern of broad-scale allozyme homogeneity between Cretan and Swedish populations of this species [6] is not the result of the lower variability of the allozyme data.

At large scales (2000–6000 km), no significant genetic differentiation is detected among sampling sites within (pairwise φ_ST_, φ_ST_, G_ST_ and φ_SC_) and between (φ_CT_) oceanographic areas, indicating that there is no mtDNA differentiation in *M. neritoides* throughout the NEA. Yet, a small amount of differentiation is detected at the intra-population level (φ_IS_), i.e. among individuals within sampling site. Intra-population variation without inter-population differentiation reflects the very high diversity of haplotypes within sampling sites, and besides, may be a sampling artefact since the Scottish population in ATCO has a smaller sample size (*N* = 18) than any other population (*N* = 32 to 43). As such, its haplotype composition may be more biased than elsewhere due to the extremely high haplotype richness of *M. neritoides* [3]. At smaller scales (1.2 km, 100 km, 550 km), our results also show no mtDNA differentiation at all among sampling sites. This was also reported at a very small scale (30 m) between upper and lower shores in Silleiro, Spain [38]. Thus, *M. neritoides* shows no sign of population genetic structure and, although we note that selection is potentially acting on *M. neritoides* mtDNA and may bias gene flow estimates by violating the assumption of neutrality which underlies the coalescent model of Migrate-n [11, 34], our results suggest that *M. neritoides* is panmictic over the entire NEA basin.

The Atlantico-Mediterranean transition (defined here as the area encompassing the Gibraltar Strait, the Almeria-Oran Front and the Siculo-Tunisian Strait) and the English Channel potentially form barriers to dispersal, and hence possible phylogeographic breaks for planktonic-dispersing species [49–51]. Yet, our study did not find any evidence of barriers to gene flow or phylogeographic breaks over the entire NEA basin.

### Hyperdiverse mtDNA and assessing gene flow

To the best of our knowledge, the present work is the first gene flow and genetic connectivity estimation in a marine gastropod over its entire geographic range in the NEA using a coalescent approach. The quantitative assessment of gene flow in *M. neritoides*, based on gene genealogies using Migrate-n, shows substantial gene flow within the whole NEA basin (*N*_*e*_*m* = 558 to 4419). This high rate of gene flow counteracts genetic drift and provokes spatio-temporal homogeneity of the species gene pool. Although global within the NEA, gene flow appears strongly directed eastward from the Atlantic towards the Mediterranean, than westward from the Mediterranean to the Atlantic. In this eastward gene flow pattern from the Atlantic to the Mediterranean, the Atlantic European coasts seem to act as a stepping-stone for gene flow from more western Atlantic areas such as the Azores. This pattern was apparent with data showing high mtDNA polymorphism (dataset A) but is no more supported with data showing reduced mtDNA polymorphism (dataset B). Therefore, model ranking in Migrate-n gene flow analyses is seemingly not influenced by the amount of mtDNA diversity, whereas model probability is influenced by the amount of mtDNA diversity and subsequent selection of one single model is better defined without mtDNA hyperdiversity.

A recent illustration of how hyperdiverse mtDNA data may affect the interpretation of genetic connectivity is provided by the Atlantic coral-dwelling crab *Opecarcinus hypostegus*. This species has a planktonic larval development (PLD unknown), with supposedly high potential for long-distance dispersal. Yet, gene flow in this species seems to be limited and follows an isolation-by-distance pattern [52]. Like *M. neritoides*, *O. hypostegus* shows an extreme degree of mtDNA COI variation (*Hd* = 0.999; *π* = 0.026; 22% polymorphic sites; *H*_*p*_ = 187 out of 195 specimens) [52]. This high mtDNA diversity was interpreted as an early sign of speciation resulting from adaptive genetic divergence over the coral host species. Yet, Fu and Li’s F and Tajima’s D were non-significant [52] and hence do not provide signal of selection and/or demographic expansion. Moreover, the nucleotide diversity at synonymous sites in *O. hypostegus* (calculated from [52]), is well-above the threshold of 5% (*π*_*syn*_ = 10.2%), indicating mtDNA hyperdiversity. This is in line with the bush-like pattern of the mtDNA haplotype network [Fig. 2 in 52] typical of mtDNA hyperdiversity, thus making the claim of cryptic species premature. Similar to our results on *M. neritoides*, mtDNA hyperdiversity in *O. hypostegus* may result from an elevated mutation rate, but unlike *M. neritoides’* mtDNA hyperdiversity which is shaped by selection, *O. hypostegus*’ mtDNA hyperdiversity may be maintained on account of limited gene flow rather than of selection suggested by the authors.

The coalescent-based gene flow rates in *M. neritoides* are very high, notably from the Atlantic European coasts to the Mediterranean Sea (*N*_*e*_*m* = 4419), comparable to other substantial long-distance gene flow rates of planktonic-dispersing species within the NEA: (1) the periwinkle *Tectarius striatus* with *N*_*e*_*m* = 18 to 290 over 1900 km among Azores, Madeira and the Canary Islands, but with very limited gene flow over 1500–2500 km between the Cape Verde Islands on the one hand, and the Azores, Madeira and the Canary Islands on the other (*N*_*e*_*m* = 3) [53], (2) the sea urchin *Paracentrotus lividus* over 3700 km within the Mediterranean (*N*_*e*_*m* = 60) and over 5000 km from the Atlantic European coasts to the eastern Mediterranean (*N*_*e*_*m* = 30) [54], and (3) the bivalve *Scrobicularia plana* over 4500 km along the Atlantic European coasts (*N*_*e*_*m* = 903) [55]. Substantial long-distance gene flow is also reported outward the NEA, for the sea cucumber *Cucumaria frondosa* over 5000 km from Norway to the East coasts of North America (*N*_*e*_*m* = 80) [56]. The PLD of 4–8 weeks in *M. neritoides* is comparable to that of *Paracentrotus lividus* (PLD = 3 weeks) [57], *Scrobicularia plana* (PLD = 2–4 weeks) [58] and *Cucumaria frondosa* (PLD = 6 weeks) [59] (the PLD of *Tectarius striatus* is unknown). This suggests that, as expected, planktonic-dispersing species with a long-lived larval dispersal stage may achieve high levels of gene flow in the NEA basin.

**Fig. 3:**
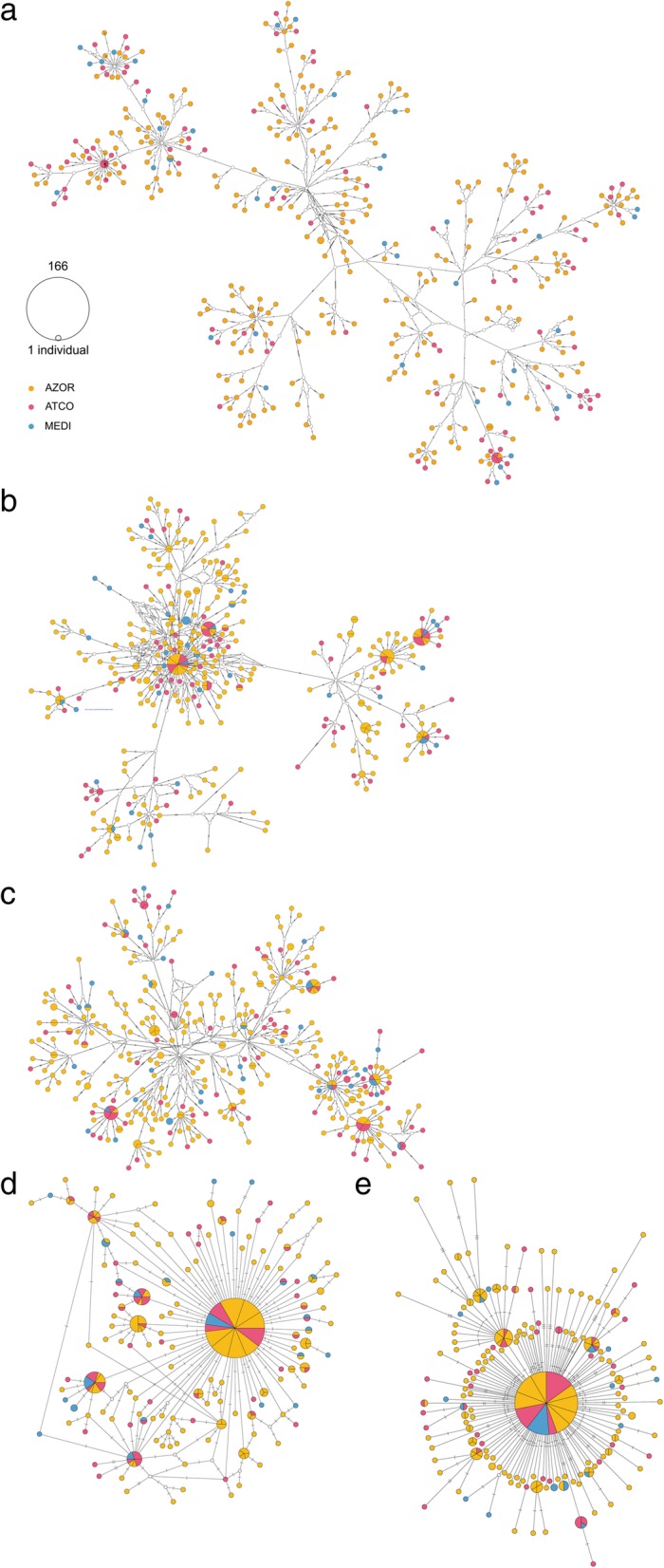
Distribution range of *Melarhaphe neritoides* (ETRS89 Lambert azimuthal equal-area projection, EPSG:3035) and 11 sites sampled. Fajã Grande, Flores island, Azores, Portugal (FLO); Varadouro, Faial island, Azores, Portugal (FAI); Lajes do Pico, Pico island, Azores, Portugal (PIC); Porto Formoso, São Miguel island, Azores, Portugal (SM1); port of Ribeira Quente, São Miguel island, Azores, Portugal (SM2); shore of Ribeira Quente, São Miguel island, Azores, Portugal (SM3); Maia, Santa Maria island, Azores, Portugal (SMA); North Berwick, Scotland, United Kingdom (SCO); Lisbon, Portugal (POR); Vigo, Spain (SPA); Kamiros Skala, Rhodes island, Greece (RHO). The arrows represent the major surface (solid line) and deep (dashed line) sea currents: Azores Current (AC); Atlantic Water Current (AWC); Canary Current (CC); Irminger Current (IC); Levantine Intermediate Water (LIW); Mediterranean Outflow Water (MOW); North Atlantic Current (NAC); North Atlantic Drift Current (NADC); Norwegian Current (NC); Portugal Current (PC); Slope/Shelf Edge Current (SC)

The directional pattern of gene flow in *M. neritoides* as described by model M6 inferred from hyperdiverse mtDNA (dataset A) is congruent with the history of the sea currents in the NEA (Fig. 3). Short-lived Pleistocene sea surface currents allowed the colonization of Macaronesia from Eastern Atlantic areas [60]. However, nowadays the Azores Current flows eastward to Gibraltar, where its surface water enters the Mediterranean through the Atlantic Water Current [61, 62], suggesting that larval transport predominantly occurs from Macaronesia towards the Mediterranean. Originating from the Gulf Stream, the North Atlantic Current [63] branches into the Irminger Current [64], the North Atlantic Drift Current [65] and the Slope/Shelf Edge Current [66], which flow northeastward through the NEA and likely transport larvae from the Azores to the Atlantic European coasts above 50°N to Iceland, the British Isles and France. The average flow of the Portugal Current is southward to Africa [67], feeding the Canary Current and also entering the Mediterranean in a shallow surface layer [68], suggesting that larval transport predominantly occurs from the Atlantic European coasts to the Mediterranean. In the opposite directions, gene flow appears weaker from the Mediterranean westward to the Atlantic European coasts and Macaronesia, as it goes against mainstream currents and rather follows the Levantine Intermediate Water and the Mediterranean Outflow Water that flow below 500 m depth westward to Macaronesia and northward to Ireland [62, 69], as well as the seasonal northward flow of the Portugal Current in winter. Therefore, the Atlantic European coasts and Macaronesia are most probably a source of new, dispersing, haplotypes supplying the Mediterranean, rather than sinks receiving new haplotypes from the Mediterranean.

## Conclusions

The mtDNA data presented here strongly suggest that *Melarhaphe neritoides* shows no genetic structure and is panmictic over its entire distribution range in the NEA, though with a predominantly eastward gene flow. The Mediterranean acts as a sink receiving large numbers of immigrants per generation from primarily the NEA coasts (*N*_*e*_*m* > 800). Direction in gene flow is, however, no more evident after removing hyperdiversity from mtDNA, suggesting a potential influence of mtDNA polymorphism on coalescent-based inference of gene flow model probability. The gene flow pattern revealed here is consistent with prior expectations and allozyme data. The mtDNA hyperdiversity (*π*_*syn*_ ≥ 5%) of *M. neritoides* results in a lack of shared haplotypes among localities sampled throughout the NEA, up to a complete haplotypic differentiation between localities as close as 1.2 km. Yet, the deceiving haplotypic mtDNA differentiation among localities does not reflect a lack of gene flow, but results from the concealed signal of gene flow by the high mutation rate, so that sampling efforts are too low to detect shared haplotypes with realistic probabilities. When using such hyperdiverse genetic markers, population genetic differentiation in terms of a lack of shared haplotypes may be substantial, but is not indicative of population genetic differentiation in terms of fixation of alternative haplotypes (D_EST_ ≈ 1 does not a fortiori imply φ_ST_ ≈ 1). Because F_ST_ (and its related parameters G_ST_, φ_ST_) and D_EST_ measure two different characteristics of population genetic differentiation, the inappropriate use of these indices can lead to erroneous interpretations of population genetic differentiation. However, when using F_ST_ (or its relatives) as a measure of population differentiation through fixation of alternative alleles according to the original recommendation of Wright [18, page 82], and D_EST_ as a measure of population differentiation by a lack of haplotype sharing as intended by Jost [26], the presence of mtDNA hyperdiversity will not affect population genetic interpretations, in which case F_ST_ (and relatives) and D_EST_ are complementary. When coalescent-based gene flow inference is not possible, combining φ_ST_ with D_EST_ gives reasonable clues about migration using hyperdiverse mtDNA.

## Methods

### Sample collection and DNA sequencing

We used 399 specimens of *M. neritoides* from 11 sampling sites throughout the species’ distribution range in the NEA (Fig. 3, Table 6). Figures 1 and 3 were created using the open source geographic information system QGIS 2.8.8 [70] and shoreline data from the “Global Self-consistent Hierarchical High-resolution Geography” database [71]. All specimens were stored at − 20 °C until DNA analysis. Remaining body parts were preserved in ethanol and deposited in the collections of the Royal Belgian Institute of Natural Sciences, Brussels (RBINS) under the general inventory number IG 32962. Genomic DNA extraction, amplification and sequencing of the 16S (482 bp), COI (614 bp) and Cyt*b* (675 bp) mtDNA gene fragments, sequence assembly and alignment, were performed as described in Fourdrilis et al. [3]. In total, 1197 sequences of 16S, COI and Cyt*b* gene fragments were used, 555 of which were previously published in Fourdrilis et al. [3] (GenBank: KT996152-KT997344), and 642 were obtained from 214 newly sequenced specimens (GenBank: KX537775-KX538416). The three gene fragments were used as single gene datasets, and were also concatenated using Geneious 5.3.4 (http://www.geneious.com, [72]), producing 399 combined 16S-COI-Cyt*b* haplotypes (1771 bp) referred to as “dataset A”.

**Table 6:** Location of sampling sites and number of *Melarhaphe neritoides* specimens sampled

Sampling site	*N*	Sampling date	WGS84 coordinates	
			Latitude	Longitude
FAI	42	06/28/1993	N 38.56632	W 28.77069
FLO	39	1992	N 39.45817	W 31.26401
PIC	37	10/14/1993	N 38.39633	W 28.25684
POR	38	08/07/2013	N 38.70514	W 9.14312
RHO	39	10/11/2011	N 36.27311	E 27.82419
SCO	18	05/28/1997	N 56.06206	W 2.71623
SM1	35	07/31/1993	N 37.82305	W 25.42695
SM2 (port)	37	06/30/2012	N 37.7350	W 25.29717
SM3 (praia)	43	06/30/2012	N 37.7295	W 25.30801
SMA	32	04/17/1996	N 36.94016	W 25.01322
SPA	39	08/06/1995	N 42.22458	W 8.76987
Total	399			

*N*, total number of sampled individuals in the present study

### mtDNA hyperdiversity and assessing population genetic differentiation and connectivity

The impact of mtDNA hyperdiversity on assessing population genetic differentiation and connectivity was investigated using two datasets: one with and one without hyperdiversity. The hyperdiverse dataset (A) contained the original, unmodified, 16S-COI-Cyt*b* data. The dataset without hyperdiversity (B) was derived from the hypervariable dataset A by removing the hypervariable nucleotide sites. To this end, the original hypervariable mtDNA data (A) were imported into Network 5.0.0.1 [73] and hypervariable nucleotide sites were identified in the *.sta* outfile as the characters showing a weight > 1, which correspond to fast-mutating nucleotide sites and/or sites segregating for two or more nucleotides (i.e. showing three or more variants). In this way, 346 hypervariable nucleotide sites were deleted from the 540 variable sites in the sequence alignment, representing respectively 10, 24 and 23% from the length of the 16S, COI and Cyt*b* gene fragments. The total length of the new multiple sequence alignment is 1429 bp. This procedure allows to preserve the high genetic diversity (*Hd* moved from 0.999 ± 0.001 to 0.824 ± 0.001) while lowering the amount of polymorphism (*S* decreased from 30 to 13% and *π* from 0.013 ± 0.001 to 0.001 ± 0.001) (Table 1).

### Population genetic diversity and differentiation analyses

mtDNA diversity metrics were computed for dataset A and dataset B in the 11 sampling sites separately and after pooling the 11 sites (referred to as “total population”), and for the three single gene datasets in the total population, using DnaSP 5.10.1 [74]. DnaSP considers sites with alignment gaps in the 16S sequences as fifth nucleotide states for the calculations of *H* and *Hd*, but excludes them from the calculations of *S*, *π*, *π*_*syn*_ and *π*_*nonsyn*_.

Population genetic differentiation was assessed in the total population (datasets A and B), by calculating G_ST_ [75] and N_ST_ based on a distance matrix of pairwise differences [46] using Spagedi 1.4 [76], φ_ST_ based on a distance matrix of pairwise differences between haplotypes [77] using Arlequin 3.5.1.3 [78], and the unbiased Morisita dissimilarity index D_EST_ using Spade [79]. Population genetic differentiation was also assessed among pairs of sampling sites by calculating pairwise φ_ST_ using Arlequin. The significance of pairwise φ_ST_ was corrected for multiple test biases using the Sequential Bonferroni procedure [80] and only *p*-values that remained significant after these corrections were considered to be meaningful. Hierarchical analyses of molecular variance [AMOVA, 77] of Tamura-Nei distances among haplotypes were performed using Arlequin, in order to quantify population genetic differentiation among groups (φ_CT_), among populations within groups (φ_SC_) and within populations (φ_IS_) at several geographic scales, and to test for panmixis. The significance of φ-statistics was assessed using 90,000 permutations of individuals among populations, and of populations among geographic groupings. A population is a sampling site. Three groupings were defined to represent the three oceanographic areas of interest (Fig. 3), i.e. the North East Atlantic coast (ATCO, *N* = 95), the remote Azores archipelago at the southwesternmost border of the distribution area (AZOR, *N* = 265), and the Mediterranean (MEDI, *N* = 39). The AMOVA with three groupings contains nine populations following a sampling scheme *k* = 5,3,1 (i.e. first grouping including five populations, second grouping including three populations and third grouping including one population) and hence provides adequate statistical power (i.e. *p*-value ≤ 0.05 and at least 20 unique permutations) at this level [81].

### Population genetic connectivity analyses

**Fig. 4:**
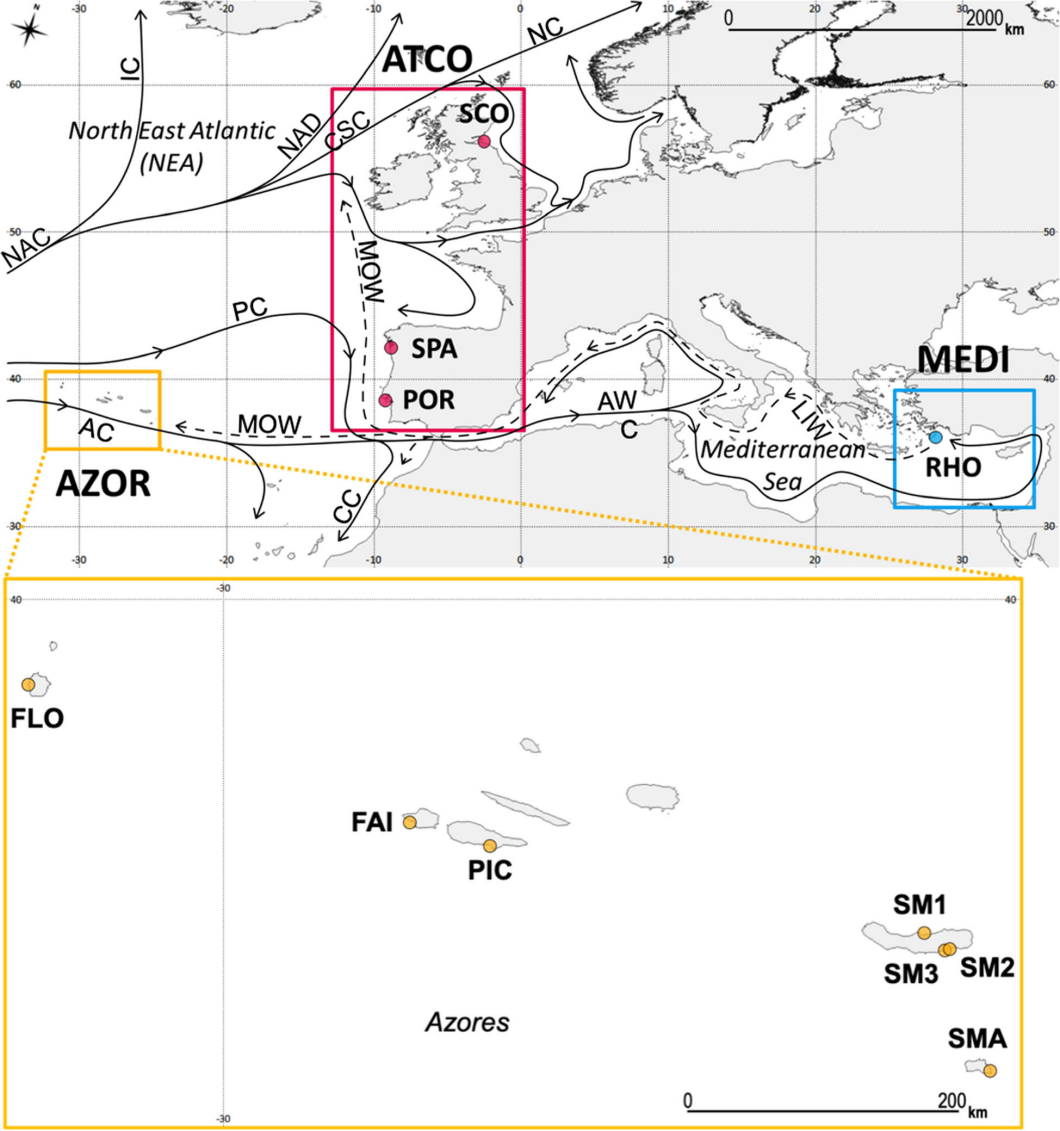
Diagrams of migration models for *Melarhaphe neritoides* larval dispersal tested in Migrate-n. (*M*) mutation-scaled immigration rate, (*θ*) mutation-scaled population sizes, (M1) full migration model, (M2) island model, (M3) source-sink “eastward” model with two sources, (M4) source-sink “westward” model, (M5) panmixia, (M6) source-sink “eastward” model with one source. Arrows represent directions of gene flow among the three oceanographic groups AZOR (Azores), ATCO (North East Atlantic coast) and MEDI (Mediterranean). (*) variable migration rate parameter, (*m*) symmetrical migration rate parameter, (0) migration rate parameter not estimated

Population genetic connectivity in *M. neritoides* was qualitatively investigated by reconstructing a median-joining haplotype network [73] using PopART 1.7 [82] on the three single gene datasets, dataset A and dataset B. Such a network provides information about phylogeographic structure and gene flow among populations. Population genetic connectivity was then assessed and compared between datasets A and B by quantifying long-term gene flow, or immigration rate (i.e. *N*_*e*_*m* the effective number of immigrants per generation), among the three oceanographic areas AZOR, ATCO and MEDI in the NEA basin, using the Bayesian MCMC method implemented in Migrate-n 3.6.11 [83] and hosted on the CIPRES Science Gateway [84]. Migrate-n estimates the mutation-scaled population size (*θ* = 2*N*_*e*_*μ* for haploid mtDNA) for each area and the mutation-scaled immigration rate (*M* = *m*/*μ*). Subsampling the three oceanographic areas to get equal sample sizes was not necessary as the difference between the largest (AZOR, *N* = 265) and the smallest (MEDI, *N* = 39) sample sizes was less than ten-fold (personal communication: Peter Beerli, School of Computational Science and Information Technology at Florida State University). Five models of dispersal were first evaluated (Fig. 4): (M1) a full migration model with three population sizes and six immigration rates, (M2) an island model where all areas share a single mean estimate of *θ* and exchange genes with all other areas at the same mean rate, (M3) a source-sink model with three population sizes and three directional West-to-East immigration rates, where the main sink is MEDI receiving immigrants from the sources AZOR and ATCO, and the second sink is ATCO receiving immigrants from AZOR, (M4) a source-sink model with three population sizes and three directional East-to-West immigration rates, where the main sink is AZOR receiving immigrants from the sources MEDI and ATCO, and the second sink is ATCO receiving immigrants from MEDI, and (M5) a panmictic model with one population size parameter. Preliminary results showed that M3 was the second best model after M5, and included the possibility of null gene flow from AZOR to MEDI. Following this observation, an additional model was tested based on the hypothesis that setting the gene flow to zero from AZOR to MEDI would best fit the data: (M6) a source-sink model like M3 with three population sizes, but only two directional West-to-East immigration rates. In M6, the two sinks MEDI and ATCO are the same as in M3, but MEDI receives immigrants from only one source (ATCO) and not from AZOR. We ran Migrate-n analyses under an F84 mutational model, with a windowed uniform prior for *θ* and *M*, the bounds of which are (0; 2) and (0; 9500) respectively. For each model, we ran three replicates using four MCMC chains with relative temperatures of 1.0, 1.5, 3.0 and 100,000, and of 500 million generations, which sampled one of every 100 iterations. The first 30% of generations were discarded from each run as burn-in. The Migrate-n analyses were computationally intensive. When replicates were run consecutively, five to 12 weeks were required depending on the model. The use of the message passing interface version of Migrate-n, enabling simultaneous analysis of replicates using three nodes, decreased computing time to 18–34 days. Convergence of MCMC chains was assessed by visual examination of the log trace of each posterior distribution showing caterpillar shape, and making sure that the effective sampling size value of each statistic was > 200 [85], using the ‘coda’ package [86] in R 3.0.2 [87]. The R script is accessible on Figshare (see Data Accessibility section). The models were ranked using log Bayes factors (LBF) and probabilities (p), that compare the marginal likelihood of each model calculated using the thermodynamic integration method implemented in Migrate-n [88]. The ranking tells how useful a model is to infer a relationship between the pattern of connectivity hypothesised and the biology of *M. neritoides*. The most useful information is found in the model ranked first. The effective number of immigrants per generation was calculated for haploid data with female-transmission following the equation *N*_*e*_*m* = *θ*_recipient_*M [89]. The effective population size was calculated with the equation *N*_*e*_ = *θ/μ* using *μ* = 1.99 × 10^− 4^ mutations per nucleotide site per generation from Fourdrilis et al. [3]. In order to verify the assumption of neutral evolution that underlies the coalescent model of gene flow inference in Migrate-n, selection was assessed by applying Fay & Wu’s H statistic [90] to dataset A using DnaSP 6.12.01 [91]. *Tectarius striatus* was the most closely related species to *M. neritoides* [92] for which the three same gene fragments of 16S, COI and Cyt*b* were available on Genbank (U46825, AJ488644, U46826), and was therefore used as outgroup for the Fay & Wu test. The 95% confidence interval was calculated based on 10,000 coalescent-based simulations.

## Abbreviations

AC: Azores Current
AMOVA: Analysis of Molecular Variance
ATCO: North East Atlantic coast
AWC: Atlantic Water Current
AZOR: Azores archipelago
bp: base pair
CC: Canary Current
CI: confidence interval
FAI: Varadouro, Faial island, Azores, Portugal
FLO: Fajã Grande, Flores island, Azores, Portugal
*H*: number of haplotypes
*Hd*: haplotype diversity
*H*_*p*_: number of private haplotypes
*H*_*s*_: number of haplotypes shared among sampling sites
*H*_*w*_: number of haplotypes shared within sampling site
IC: Irminger Current
*L*: DNA fragment length in base pairs
LBF: log Bayes factors
LIW: Levantine Intermediate Water
*M*: mutation-scaled immigration rate
M1: full migration model
M2: island model
M3: source-sink eastward
M4: source-sink westward
M5: panmixia
M6: source-sink eastward
MCMC: Markov chain Monte Carlo
MEDI: Mediterranean
MOW: Mediterranean Outflow Water
mtDNA: mitochondrial DNA
*N*: number of individuals
NAC: North Atlantic Current
NADC: North Atlantic Drift Current
NC: Norwegian Current
NEA: North East Atlantic
PC: Portugal Current
PIC: Lajes do Pico, Pico island, Azores, Portugal
PLD: pelagic larval duration
POR: Lisbon, Portugal
RBINS: Royal Belgian Institute of Natural Sciences
RHO: Kamiros Skala, Rhodes island, Greece
*S*: number of segregating sites
SC: Slope/Shelf Edge Current
SCO: North Berwick, Scotland, United Kingdom
SD: standard deviation
SM1: Porto Formoso, São Miguel island, Azores, Portugal
SM2: port of Ribeira Quente, São Miguel island, Azores, Portugal
SM3: shore of Ribeira Quente, São Miguel island, Azores, Portugal
SMA: Maia, Santa Maria island, Azores, Portugal
SPA: Vigo, Spain
