# Correction to: Highly polymorphic mitochondrial DNA and deceiving haplotypic differentiation: implications for assessing population genetic differentiation and connectivity

**DOI:** 10.1186/s12862-019-1428-x

**Published:** 2019-05-17

**Authors:** S. Fourdrilis, T. Backeljau

**Affiliations:** 1 Royal Belgian Institute of Natural Sciences, Rue Vautier 29, B-1000 Brussels, Belgium; 2 Evolutionary Ecology Group, University of Antwerp, Universiteitplein 1, B-2610 Antwerp, Antwerp, Belgium

**Correction to: BMC Evolutionary Biology, 2019; 19:92**

**DOI: 10.1186/s12862-019-1414-3**

After publication of the original article [1], the authors have notified us that the order of the figures was incorrect. The figure captions were all correctly placed, however the figures themselves had to be offset by 1 position:

- Fig. 1 had to be replaced by originally published Fig. 2
- Fig. 2 had to be replaced by originally published Fig. 3
- Fig. 3 had to be replaced by originally published Fig. 4
- Fig. 4 had to be replaced by originally published Fig. 1

The original article has been corrected.
